# Associations between School Food Environments, Body Mass Index and Dietary Intakes among Regional School Students in Victoria, Australia: A Cross-Sectional Study

**DOI:** 10.3390/ijerph16162916

**Published:** 2019-08-14

**Authors:** Laura Alston, Nicholas Crooks, Claudia Strugnell, Liliana Orellana, Steven Allender, Claire Rennie, Melanie Nichols

**Affiliations:** 1 Global Obesity Centre (GLOBE), Faculty of Health, Deakin University, Geelong 3220, Victoria, Australia; 2 Biostatistics Unit, Faculty of Health, Deakin University, Geelong 3220, Victoria, Australia; 3 Cancer Council Victoria, Melbourne 3004, Victoria, Australia

**Keywords:** childhood overweight, obesity, school food environment, dietary intakes, regional

## Abstract

(1) Background: Childhood overweight and obesity is a significant and preventable problem worldwide. School environments have been suggested to be plausible targets for interventions seeking to improve the quality of children’s dietary intake. The objective of this study was to determine the extent to which the current characteristics of the school food environment were associated with primary school students’ dietary intake and Body Mass Index (BMI) z scores in a representative sample in regional Victoria. (2) Methods: This study included 53 schools, comprising a sample of 3,496 students in year levels two (aged 7–8 years), four (9–10 years) and six (11–12 years). Year four and six students completed dietary questionnaires. Principals from each school completed a survey on school food environment characteristics. Mixed-effects logistic regression was used to assess the relationship between students’ dietary intake and school food environment scores, controlling for confounders such as socio-economic status, school size and sex. Food environment scores were also analysed against the odds of being healthy weight (defined as normal BMI z score). (3) Results: Mixed associations were found for the relationship between students’ dietary intake and food environment scores. Meeting the guidelines for vegetable intake was not associated with food environment scores, but students were more likely (OR: 1.68 95% CI 1.26, 2.24) to meet the guidelines if they attended a large school (>300 enrolments) and were female (OR: 1.28 95% CI: 1.02, 1.59). Healthy weight was not associated with school food environment scores, but being a healthy weight was significantly associated with less disadvantage (OR: 1.24 95% CI 1.05, 1.45). Conclusion: In this study, the measured characteristics of school food environments did not have strong associations with dietary intakes or BMI among students.

## 1. Introduction

Childhood overweight and obesity is a preventable, yet persistent and significant problem worldwide [1]. There is ample evidence to show the health impacts of childhood overweight and obesity throughout childhood, adolescence and into adulthood [1,2].

Multiple factors across many levels of food environments work against obesity prevention in childhood, including heavy marketing of unhealthy food options to children, high availability of cheap and calorific foods, social norms and environments that encourage an over-consumption of food [1]. Although there are many family environment characteristics that can influence a child’s dietary intake, such as parental dietary habits and practices [3,4], children spend a large portion of time at school between the ages of 5 and 18 years [5], making this an important environment to understand and potentially optimise.

The school food environment is considered to include all food and drink that is available or provided to students attending the school, whether this be through the school canteen, fundraising activities or food provision programs (e.g., breakfast programs) [6,7]. The school food environment is influenced by social norms and the presence or absence of government and/or local school policies [6,7].

Interventions targeting the school food environment have shown potential in influencing both eating behaviours and body mass index (BMI) of students in the United States of America (USA) and United Kingdom (UK) [6]. Subsequently, schools are viewed as one of the key environments in which to tackle childhood obesity [6,7,8]. A review of school food environment interventions found that 17 out of 18 studies showed a significant increase in students’ healthy eating behaviours or a decrease in mean body mass index (BMI), although the review authors noted that only two of the included studies were assessed to be methodologically of high quality [7]. The review assessed a range of interventions including reductions in the portions and availability of discretionary items (such as chips, chocolate, or food that is not necessary for health) [8], increases in the amount of fruit and vegetables available through the school canteen [9], and changes to policy around the provision of school lunch programs [7,10]. Conversely, there is evidence to show that reducing the availability of discretionary food items does not always guarantee increased consumption of fruit and vegetables [11]. A study by Taber et al. showed that restricting items such as sugar-sweetened beverages (SSBs) in the school food environment reduces consumption among middle school students, but does not always translate to reductions in overall consumption of SSBs [12].

Cross-sectional research has also shown that restricting access to discretionary foods in the school food environment may encourage lower calorie intake and improved BMI [13,14] in the USA and UK, and the presence of vegetable gardens has been associated with improved fruit and vegetable intakes among students [15]. Cross-sectional studies in the USA have shown mixed associations between the school food environment, dietary intakes and unhealthy BMI. Fruit and vegetable intake and availability of these foods within the school food environment was minimally associated [16], yet the caloric intake from discretionary foods obtained at school was significantly associated with obesity [14,17]. 

This is the first cross-sectional study in Australia to use a census-styled school recruitment process (all primary schools invited) with a high participatory opt-out approach for students and measured height and weight. This is the first Australian study to also provide an assessment of multiple primary school food environment components and the association with dietary intake, as well as odds of healthy weight compared to overweight and obesity of children attending these schools.

The objectives of this cross-sectional analysis in a large sample of schools in regional Victoria, Australia, are to

- Determine what school-level characteristics are associated with having low, medium and highly scored school food environments.
- Understand associations between characteristics of the school food environment with school students’ dietary intake in primary schools, with adjustment for confounders such as socioeconomic status (SES), sex and school size.
- To assess the strength of associations between the healthiness of the school food environment and with the level of overweight and obesity within a school with adjustment for SES, sex and school size.

## 2. Materials and Methods

### 2.1. Sampling

The data used for this study were collected for two separate studies in the state of Victoria, being the WHOSTOPS study [18] and Goulburn Valley Health Behaviours Monitoring study (GVHBMS) [19]. The WHOSTOPS study involved six local government areas (LGAs) in South West Victoria, and the GVHBMS involved three LGAs in North Eastern Victoria, all of which are classified as either inner or outer regional areas by the Australian Geographical Standard [20]. While they are located in very different geographical regions, these studies employed the same sampling and data collection methods, described in detail previously [21]. In brief, all primary schools in both study regions were invited via a letter to the principal and participated between July 2016–June 2017. All Year 2 (aged approx. 7–8 years), 4 (aged approx. 9–10 years) and 6 (aged approx. 11–12 years years) students at participating schools were invited to take part. Both studies used an opt-out approach whereby students who did not want to participate returned an opt-out form, signed by their parents or guardians. The study team visited each school to conduct the anthropometric (height and weight) and behavioural measures (self-reported questionnaire) from the students and the school environment audit from the school principal or a delegate. 

(1) Anthropometric Measures (Year 2, Year 4 and Year 6): 

The measured height and weight of students were taken by trained staff in a private measuring booth to ensure students maximum privacy. Height was measured to the nearest 0.1 cm using a Portable Stadiometer (Charder HM 200P Portstad, Charder Electronic Co Ltd, Taichung City, Taiwan). Weight was measured to the nearest 0.05 kg, using Digital weight scales (A&D Precision Scale UC 321, A7D Medical, San Jose, CA, USA). To ensure accuracy, all students were measured twice and where the two initial measures differed by more than 0.5 cm and 0.1 kg for height and weight, respectively, a third measurement was taken. The mean of all values was used in the analysis.

Student’s measured height and weight was used to calculate BMI z score, which was dichotomised as ‘healthy weight’ (normal BMI z score) or ‘overweight and obese’ using the World Health Organization (WHO) guidelines [22]. We removed data for students classified as ‘underweight’ due to very small numbers (n = 17), and this did not alter the outcome of the analysis. 

(2) Dietary questionnaires (Year 4 and Year 6):

Students individually completed electronic questionnaires on Samsung Galaxy tablets, with guidance from a trained session supervisor, which took approximately 30–35 min (see Supplementary Material). A dietary questionnaire (a simple food frequency questionnaire, validity and reliability published by Hayward et al. [23]) covering the daily consumption of fruit, vegetables and daily water along with weekly dietary habits around the consumption of SSBs and discretionary foods such as packaged snacks and take away foods.

Diet was summarized as dichotomised variables, as guided by the Australian Guide to Healthy Eating [24], indicating whether or not the student reported average daily intakes meeting vegetable (5 serves per day for all 9–11-year-olds and girls aged 12–13 years and 5.5. for boys aged 12–13 years), fruit (2 serves per day) [24] and water (5 < glasses per day), as well as lower takeaway consumption frequency (once per week or less), and SSB consumption (less than once per day).

(3) School environment audit: 

A school environment audit was completed by the school principal or a representative on the day of data collection. This tool comprised selected questions from the Be Active Eat Well school environment audit and the validated ISCOLE school environment audit tool [25]. Questions covered both the school physical and policy environments. Physical food environment questions included the presence of school vegetable gardens, cooking classes, breakfast programs and healthy canteen programs and is available in the Supplementary Material.

**Figure 1:**
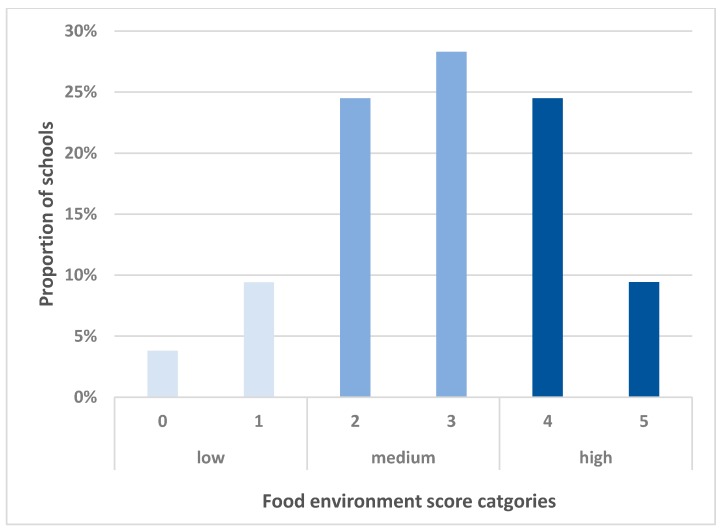
Proportion of schools with school food environment scores of 0–5.

Notes: School food environment scores reflect the number of healthy school environment characteristics reported in the school. Higher scores indicate a healthier school food environment.

A school environment score was derived as the total number of school policy and food environment features (from 0–5), based on the reported presence of; nutrition policy or practice (e.g., a regular activity within school but no formal written policy, as an example, regular fruit break per day within a primary school), a school vegetable garden, cooking classes, a breakfast program, and a healthy canteen program. For example, if a school had both a vegetable garden and a healthy canteen initiative, but no other reported healthy characteristics, the school was assigned a score of ‘2’. Scores were then categorized into three groups. ‘High’ food environment scores include schools with a score of 4–5, ‘Medium’ includes scores of 2–3, and ‘Low’ was scores 0–1 (see Figure 1). A ‘high’ school food environment score indicated a high level of health-promoting food environment characteristics. 

(4) Other school characteristics 

School socio-economic status was measured through the Index of Community Socio Educational Advantage (ICSEA) [26], which assigns an overall score, based on the socio-economic background of the students attending that school. The ICSEA score includes variables such as the proportion of Aboriginal and Torres Strait Islander students, geographical location of the school, the parental occupation and education levels of the students attending the schools [26]. ICSEA scores were dichotomised as equal or above 1000 or less than 1000, which corresponds to the national average [26]. The size of the school was categorised based on enrolments using categories from the Australian Education Act 2013 [27] (small (1–100 students), medium (200–300 students) or large (>300 students).

### 2.2. Ethical Approval

This project was approved by the Deakin University Human Research Ethics Committee (DUHREC 2014-279), the Victorian Department of Education and Training (DET 2015_002622) and the Catholic Archdioceses of Sandhurst and Ballarat.

### 2.3. Statistical Analysis

To compare the proportion of students meeting recommendations by sex, chi squared (Chi2 hypothesis tests were used. Chi squared tests were also used to examine differences in low, medium and high school food environment scores by school-level characteristics. Univariate mixed-effects logistic regression models were used to estimate the associations between the school food environment score (low, medium, high) and students’ (a) dichotomised dietary intake variables and (b) overweight/obesity. Six dietary intake variables were considered; meeting (or not meeting) recommendations for (1) vegetables intake, (2) fruit intake; and frequency of consuming (3) takeaway foods, (4) snack foods, (5) SSBs and (6) water. We also fitted mixed logistic models including adjustment for both student-level and school-level characteristics, similar to other published studies on this topic [16,17] including school socio economic status (measured by ICSEA), enrolment size (small, medium, large) and participating students’ sex (proportion of male students). All models included school as a random effect to account for the nesting (or clustering) of students within schools. Data was originally collected from 75 schools, and schools that had fewer than 10 students’ measurement data (height and weight) were excluded from this analysis due to small sample size (n = 22 schools). We defined a strong association of the models at a p value ≤ 0.05. 

## 3. Results

### 3.1. Sample Demographics and School Food Environment Charactertistics

Of all the schools and students approached for the study, 77% of students and 70% of schools participated. The final sample included 53 schools in Victoria, with a total of 3496 participating students (Table 1). Most schools were classified as ‘small’ (58%), the majority were government schools (86%), and 62% had an ICSEA score below the national average for Australia, indicating greater disadvantage. More than half (55%) of the schools reported having some form of nutrition policy or practice in place, 71% included cooking classes in their curriculum, and over 90% reported they had a vegetable garden at the school. Almost half (46%) reported they provided a breakfast program for their students and 38% offered a healthy canteen program. In this sample, 34% (n = 1121) of the students were measured to have a BMI z score in the overweight and obese category. The proportion of students with overweight or obesity was 33.4% (n = 560) among males and 33.9% (n = 561) among female students. 

Table 2 shows the proportion of students meeting recommendations for daily vegetables, fruit and water consumption and reporting a low frequency of takeaway, snack foods and SSBs. Females reported more favorable dietary intakes than their male counterparts and were significantly more likely to meet recommendations for all of the dietary measures except water intake. Females were more likely to meet vegetable guidelines—with 18.5% meeting guidelines, compared to 14.9% of male students (p = 0.02). A much higher proportion of students reported meeting fruit guidelines (73.4% of males and 79.4% of female students), having takeaway less than once per week (86.9% of males, 92.3% of females) and having SSBs less than once per day (80.5% and 85.2% of males and females, respectively).

### 3.2. School Food Environment Scores and Their Relationship to School Characteristics 

Figure 1 shows the distribution of school food environment scores. The score range was 0–5, which was subsequently categorised into ‘low’ (0–1), ‘medium’ (2–3) and ‘high’ (4–5). The majority (53%) of the schools had a ‘medium’ food environment score.

Table 3 shows the school characteristics by school food environment score categories. There were significant differences in food environment scores by ICSEA, with schools with lower ICSEA scores more likely to have a higher food environment score. Lower SES schools (lower ICSEA score) made up a higher proportion (39.4%) of schools with a high environment score, compared to less disadvantaged (25.0%) (p = 0.001). Small schools made up the majority of the sample, but large schools were significantly more likely to fall into the high scoring food environment category (44.4%) than the other sized schools (p = 0.001). 

Odds ratios estimated under unadjusted and adjusted mixed-effects logistic models are shown in Table 4 and Table 5. No strong associations between school environment score and dietary intakes indicators were found in the unadjusted or adjusted analyses, with the exception of a medium score and meeting water recommendations in the unadjusted model, and a medium score and a lower frequency of takeaway consumption in the adjusted model.

Overall, in the multivariate modelling, less school level socio-economic disadvantage, being female and attending a larger school appeared to have a stronger influence on the probability of students meeting guidelines for dietary intake.

(1) Dietary intake

In the fully adjusted models, students in larger schools (OR: 1.68, 95% CI: 1.26, 2.24) and females (OR: 1.28, 95% CI: 1.02, 1.59) were more likely to report vegetable intakes that satisfy the Australian dietary guidelines [24]. Being a girl (OR: 1.36 95% CI 1.12, 1.66) and attending a school with a higher ICSEA score (OR: 1.44, 95% CI 1.14, 1.78) was strongly associated with meeting fruit consumption recommendations. 

Students in schools with low and medium food environment scores were less likely to report a low frequency of takeaway consumption (once per week or less) and having SSBs less than once per day, when compared to students in schools with high food environment scores (reference group). However, these associations were not strong. Students from higher SES schools were significantly more likely to report a lower consumption of takeaway foods (OR: 2.28, 95% CI: 1.58, 3.28). Meeting water consumption recommendations was not significantly associated with SES, school size, or sex. The odds of meeting daily water consumption recommendations were highest in schools with a medium food environment score.

(2) Weight status

Table 4 shows no strong associations between weight status and school food environment scores in the univariate model. Table 5 shows the multivariate model for the association between weight status and school environment scores, with adjustment for SES, school size and sex. School environment scores were not associated with the odds of being within a healthy weight range (normal BMI z score) compared to overweight/obese within schools, and the ICSEA score had the strongest association (OR 1.24, 95% CI: 1.05,1.45) with being a healthy weight. 

## 4. Discussion

This cross-sectional study found that the aspects of the school food and nutrition policy/practice environment measured in this study in regional Victoria do not appear to be strongly associated with students’ dietary intakes or weight status. Although some of the intake variables did show higher odds for students attending schools with higher school food environment scores, particularly for takeaway and SSB consumption, these associations were weak.

Higher socio-economic status (SES) and female sex were much more strongly associated with optimal intakes of vegetables, fruit, take away foods, snacks and SSBs than the scored healthiness of the school food environment. This is consistent with other findings internationally, highlighting that there are multiple, interacting influences on individual dietary intakes, of which the school food environment is only one contributing factor [3,4,16,17,28,29]. Similar to findings here, measures of SES and sex, are found to be strongly associated with student’s dietary intakes and BMI than measures of the school food environment [4,16,17]. Another recent (2017) cross-sectional survey of more than 1000 primary school students in Italy showed that self-reported consumption of unhealthy foods was strongly associated with high levels of socio-economic disadvantage, with the opposite being true for self-reported consumption of healthy foods [29]. Conversely, another study found that socio-economic status was not associated with a self-reported healthier diet [30].

This study found that female students were significantly more likely to meet dietary recommendations, compared to their male counterparts. This suggests that programs, education and/or policy implemented to improve student’s dietary intakes, may benefit from having additional elements to target male students who appear to be more vulnerable to having sub-optimal diets.

All states in Australia have policies (voluntary or mandated) that can influence the school food environment, such as healthy canteen policies that encourage implementation of the ‘traffic light’ guidelines [31]. These guidelines include benchmarks on the proportion of red (unhealthy), amber (intermediate) and green (healthy) category foods that should be provided by school canteens. Despite the presence of voluntary policy to guide implementation of healthy school canteen programs in Victoria [32], previous research has shown that compliance of school canteens to these guidelines is low [33]. A study of 106 school canteen menus collected from across Victoria found that no school canteen menus met the voluntary policy guidelines, with unhealthy food items dominating school menus, and healthy foods accounting for only 20% of the food offered by the school canteen [33]. This could have implications for the present study as, although school principals reported that their school had a ‘healthy canteen program’, it has not been externally evaluated, and is likely to represent personal or organisational interpretations of what constitutes a healthy canteen program. In a similar context, a study investigating changes in the dietary intakes of student after a region-wide adoption of school nutrition policy showed marked improvements in dietary intakes, even though the schools had previously identified as having implemented nutrition policy prior to the implementation of a standard policy [34]. 

There are international examples of mandates on the provision of healthy food in schools that aim to protect against the effects of disadvantage, for example, the implementation of federally funded lunch programs in the United States of America, such as the National School Lunch Program [14]. A study evaluating the national program showed that the provision of healthy school breakfast and lunch programs improved the quality of students’ diets, especially those from lower socio-economic backgrounds; acting as protection for disadvantaged students [28]. Similar programs, focused on disadvantaged Australian students may add significant value to school food environments and in part, act as protection against obesity and diet-related disease development among vulnerable groups. 

Our study had similar findings to cross-sectional research conducted in Canada that investigated associations between dietary intake and school food environment aspects (such as the presence of policies, programs, availability) and found no associations between intake and the food environment, except for SSBs [17]. That study found a significant association between a high availability of SSBs in the school and increased SSBs consumption among students [17]. In contrast, another cross-sectional study of similar sample size in the United States, found that the presence of school policies and practices that focused on limiting energy-dense foods showed significant associations with students’ reported intake of discretionary food [24], such as fried chips and SSBs [14]. Notably, our findings in the multivariate analysis showed that students experiencing high levels of socio-economic disadvantage and boys were the most vulnerable to sub-optimal dietary intakes, consistent with previous research [17,28].

These findings highlight the difficulty in overcoming the systemic drivers of overweight/obesity, particularly socioeconomic position, and the importance of developing new policies, practices and interventions to comprehensively improve school food environments which have previously offered promise in preventing childhood obesity.

**Limitations and strengths**

There are a number of limitations of this study. Firstly, the cross-sectional nature of this study limits our ability to make inference on the direction of the associations. It might be that the school environment has been improved in response to school community concerns about students’ dietary intake and/or levels of overweight and obesity. Secondly, there are measurement errors associated with the self-report nature of dietary intake data, which relied solely on the students accurately interpreting the questions and recalling their usual intake. Questions eliciting data on dietary intake also did not ask specific questions on whether students obtained and consumed food from the school grounds; students were asked about usual dietary intake only. The survey of the school food environment was also completed by each school principal, measurement errors related to self-report data would still apply. In addition, the survey did not elicit more detailed information about the school food environment components, for example, the questionnaire did not interrogate additional information on the quality of the school vegetable garden, how regularly students are encouraged to interact with it, or how strongly it is integrated into the curriculum. There are multiple strengths of this study, including that it is based on a large sample of students and schools, covering nine local government areas of Victoria, and is representative of the schools and students in those regions.

## 5. Conclusions

This cross-sectional study provides evidence that the current school policy/practices and food environment in Victorian schools does not appear to be strongly associated with the dietary intake or BMI of students attending those schools, similar to findings across the globe. Our results suggest it may be important to focus on how the school food environments can address sub-optimal dietary intakes and obesity, particularly in more disadvantaged schools and among male students in Victoria.

## Figures and Tables

**Table 1:** School level and food environment and policy characteristics by school.

Variable	Schools (n)	Schools (% of Sample)	Students (n)	Students (% of Sample)
**Total**	53	100	3496	100
**School size**				
**Small (≥11 to ≤200 students)**	31	58.5	1086	31.0
**Medium (>200 to <300 students)**	13	24.5	964	27.6
**Large (≥300 students)**	9	17.0	1446	41.4
**School socio economic status**				
**ICSEA ^1^ < 1000**	33	62.3	2231	64.0
**ICSEA > 1000**	20	37.7	1265	36.0
**Reported presence of healthy policy and food environment components**				
**Nutrition policy or practice**	28	54.9	1638	48.4
**Cooking classes**	37	71.2	2464	73.3
**Vegetable garden**	48	90.6	3231	92.4
**Breakfast program**	23	46.0	1476	46.7
**Healthy canteen program**	17	37.8	1639	53.1

Notes: ^1^ ICSEA: Index of 1000 is the national average for a measure of socio-economic status schools with an ICSEA score of below 1000 indicating higher disadvantage, higher than 1000 indicates less disadvantage Abbreviations: ICSEA; Index of Community Socio Educational Advantage.

**Table 2:** Proportion of all Year 4 and Year 6 students meeting daily recommendations for fruit, vegetable and water consumption and consuming low frequencies of takeaway, snacks and sugar-sweetened beverages, by sex.

	Males (%)	Females (%)	P Value (Chi2)	Total (%)
**Met vegetable guidelines**	14.9	18.5	0.02	16.6
**Met fruit guidelines**	73.4	79.4	0.01	76.4
**Takeaway less than once per week**	86.9	92.3	<0.001	89.6
**Snack foods once per day or less**	51.8	57.0	0.01	54.3
**SSBs less than once per day**	80.6	85.3	0.03	82.9
**Met daily water consumption guidelines**	56.3	58.8	NS	57.5

Notes: Abbreviations Chi2; Chi squared test.

**Table 3:** Proportion of schools with low, medium and high food environment scores based on school-level characteristics.

School Characteristics	School Food Environment Scores			P Value (Chi2)
	Low % (n)	Medium % (n)	High % (n)	
**SES (ICSEA)**				
Lower ICSEA	9.0 (3)	51.5 (17)	39.4 (13)	
Higher ICSEA	20.0 (4)	55.0 (11)	25.0 (5)	<0.001
**School size (enrolments)**				
Small	12.9 (4)	54.8 (17)	32.2 (10)	
medium	15.4 (2)	53.9 (7)	30.7 (4)	
large	11.1 (1)	44.4 (4)	44.4 (4)	<0.001

Notes: low score (0–1) medium (2–3) and high (4–5). SES; socio-economic status, ICSEA; Index of Community Socio Educational Advantage.

**Table 4:** Unadjusted associations between students’ self-reported vegetable, fruit, takeaway, sugar sweetened beverages and water consumption and school food environment scores.

	Odds Ratio (95% Confidence Interval)						
	Meeting Vegetable Recommendations	Meeting Fruit Recommendations	Takeaway Less Than Once Per Week	Snack Foods Once Per Day or Less	SSBs Less Than 1x Day	Meeting Daily Water Consumption Recommendations	Healthy BMI
**School environment score (p value for overall model)**	P = 0.88	P = 0.06	P = 0.22	P = 0.83	P = 0.88	P = 0.04	
High (reference)	1.0	1.0	1.0	1.0	1.0	1.0	1.0
Medium	1.06 (0.77, 1.47)	1.29 (0.98, 1.69)	0.69 (0.4, 1.06)	1.01 (0.78, 1.31)	0.89 (0.53, 1.50)	1.20 * (0.93, 1.55)	0.85 (0.73, 1.00)
Low	0.98 (0.59, 1.57)	1.55 * (1.02, 2.37)	0.89 (0.45, 1.06)	0.91 (0.61, 1.34)	1.00 (0.45, 2.19)	0.77 (0.54, 1.12)	0.87 (0.68, 1.12)

Notes: low score (0–1) medium (2–3) and high (4–5). High score was used as the reference (1.0). * denotes a P value of <0.05 indicating a strong association Definition of meeting recommendations for vegetables (5 serves per day for all 9–11-year-olds and girls aged 12–13 years and 5.5. for boys aged 12–13 years), fruit (2 or more serves per day) and water (more than 5 glasses) refers to daily consumption. Healthy body mass index (BMI) was defined by ‘normal BMI z score’ as per World Health Organization (WHO) guidelines.

**Table 5:** Adjusted associations between students’ self-reported vegetable, fruit, takeaway, sugar-sweetened beverages and water consumption and school food environment scores.

	Odds Ratio (95% Confidence Interval)						
	Meeting Vegetable Recommendations	Meeting Fruit Recommendations	Takeaway Once Per Week or Less	Snack Foods Once Per Day or Less	SSBs Less Than 1x Day	Meeting Daily Water Consumption Recommendations	Healthy BMI
**School environment score**	P < 0.001	P < 0.001	P < 0.001	P = 0.02	P < 0.001	P = 0.13	P = 0.02
High (reference)	1.00	1.00	1.00	1.00	1.00	1.00	1.0
Medium	1.12 (0.86, 1.48)	1.21 (0.95, 1.55)	0.66 (0.46, 0.94) *	0.92 (0.54, 1.16)	0.64 (0.37, 1.07)	1.21 (0.94, 1.56)	1.09 (0.78, 1.30)
Low	0.94 (0.61, 1.43)	1.33 (1.13, 1.82)	0.70 (0.46, 0.95)	0.79 (0.72, 1.20)	0.77 (0.55, 1.08)	00.78 (0.54, 1.13)	1.01 (0.92, 1.28)
**SES (ISCEA)**							
Low ICSEA	1.00	1.00	1.00	1.00	1.00	1.00	1.00
High ICSEA (reference)	1.26 (0.96, 1.63)	1.44(1.14, 1.78) *	2.28 (1.58, 3.28) *	1.24 (0.98, 1.58)	3.30 (2.31, 4.72) *	1.04 (0.81, 1.32)	1.24 (1.05, 1.45) *
**School size**							
Small (reference)	1.00	1.00	1.00	1.00	1.00	1.00	1.00
Medium	1.13 (0.83, 1.56)	1.01 (0.77, 1.32)	1.17 (0.80, 1.72)	1.00 (0.76, 1.32)	1.41 (0.96, 2.05)	0.88 (0.67, 1.15)	0.98 (0.81, 1.18)
Large	1.68 (1.26, 2.24) *	1.14 (0.88, 1.47)	1.20 (0.82, 1.75)	0.77 (0.59, 1.01)	0.92 (0.64, 1.31)	1.08 (0.82, 1.42)	0.85 (0.72, 1.02)
**Sex**							
Boys (reference)	1.00	1.00	1.00	1.00	1.00	1.00	1.0
Girls	1.28 (1.02, 1.59) *	1.36 (1.12, 1.66) *	1.80 (1.35, 2.36) *	1.05 (1.05, 1.48) *	1.36 (1.08, 1.70) *	1.11 (0.93, 1.32)	0.97 (0.84, 1.12)

Notes: All models included school as a random effect. * denotes P < 0.05, indicating a strong association. Abbreviation: SES; socio-economic disadvantage. ICSEA; Index of Community Socio Educational Advantage. Odds of healthy BMIz score compared to overweight/obesity. High score was used as the reference (1.0). Definition of meeting recommendations for vegetables (5 serves per day for all 9–11 year olds and girls aged 12–13 years and 5.5. for boys aged 12–13 years), fruit (2 or more serves per day) and water (more than 5 glasses) refers to daily consumption. Healthy BMI was defined by ‘normal BMI z score’ as per WHO guidelines.

## Data Availability

The data are held within Deakin University and due to ethical constraints cannot be shared.

## Supplementary Materials

The following are available online at https://www.mdpi.com/1660-4601/16/16/2916/s1, Survey questions used to assess the school food environment characteristics.
