# Supplementary material for: Associations between School Food Environments, Body Mass Index and Dietary Intakes among Regional School Students in Victoria, Australia: A Cross-Sectional Study
Source: Int J Environ Res Public Health. 2019 Aug 14;16(16):2916. doi: 10.3390/ijerph16162916 (PMC6720309; doi:10.3390/ijerph16162916)
Supplement: Supplementary file 1 [file ijerph-16-02916-s001.pdf]

Primary School – Environmental Audit

Complete the following questionnaire ideally by: the school principal, a physical education teacher, or a member of the school food service.

SECTION A: SCHOOL DEMOGRAPHICS

A1. Date (dd/mm/yyyy): ______/______/_______

A2. School Name:
____

A3. School Postcode: _______________

A4. What is the position of the staff member taking part in this interview?

School Principal ☐
Vice Principal ☐
P/E Teacher ☐
Food service ☐
Other ☐

A5. School Year Level (e.g. Primary, Prep-12):
____

A6. School Gender (e.g. Co-ed, All girls, All boys):
____

A7. School Type (e.g. Government, Catholic, Independent):
____

A8. What is the total number of students in your school? (Please estimate) _____ students

A9. Does your school have trained PE/Sport specialist teachers?_________________________

SECTION **B: POLICIES AND PRACTICES**

For the following section, "policies" refers to any mandates issued by the local school board, including policies developed by your school, that affects your school environment and that have been officially adopted by your school or region. This section also asks about practices (what

your students and staff are allowed to do on a regular basis) that you might follow to promote the health and well-being of students.

B1. Does your school have written policies or practices concerning physical activity?

Yes, existing written policies ☐
Yes, written policies still under development ☐
Yes, practices ☐
No ☐
N/A ☐

B2. How effective have the policies been on overall promotion of physical activity of students in school time?

Very Effective ☐
Moderately Effective ☐
Not Effective ☐

B3. Does your school have written policies or practices concerning healthy eating of students in school time?

Yes, existing written policies ☐
Yes, written policies still under development ☐
Yes, practices ☐
No ☐
N/A ☐

B4. How effective have the policies been on overall healthy eating?

Very Effective ☐
Moderately Effective ☐
Not Effective ☐

B5. Does your school have a committee that oversees or offers guidance on the development of policies and practices concerning physical activity and healthy eating at your school (e.g., health action team, school health or wellness council)?

Yes, both physical activity and healthy eating ☐
Yes, physical activity only ☐
Yes, healthy eating only ☐
No ☐

SECTION C: PHYSICAL ACTIVITY ENVIRONMENT

C1. On average, how many hours a week are devoted to formal physical education classes for the following year levels:

Year 2 _______ hours ________minutes / per week
Year 4 _______ hours ________minutes / per week
Year 6 _______ hours ________minutes / per week

C2. On average, how many hours a week are devoted to organised sports (sport education) (e.g. netball, soccer, swimming, athletics) for the following year levels:

Year 2 _______ hours ________minutes / per week
Year 4 _______ hours ________minutes / per week
Year 6 _______ hours ________minutes / per week

C3. Compared to the class time allotted to physical education (PE)/sport education as mandated do students in your school receive on average:

Less than the mandated amount ☐
Approximately the mandated amount ☐
More than the mandated amount ☐
No specific amount is mandated ☐

*NB: Victoria mandates that all students in year P-3 get 30mins/day and students in year 4-6 get 3hrs/week (minimum of 50% PE) of physical education (PE)/sport education

C4. From the following list, please indicate which sports are offered in your school sports program (e.g. interschool and intraschool sports):

Basketball	☐	AFL	☐	Soccer	☐
Baseball	☐	Rugby	☐	Netball	☐
Gymnastics	☐	Track & Field	☐	Swimming	☐
Dance	☐	Cricket	☐	Hockey☐	

Other____

C5. To the best of your knowledge, how well do each of the following statements characterize your school?

	A lot	Sometimes	Very little	Not at all
We use physical activity as a reward	☐	☐	☐	☐
We promote physical activity during or as part of special events	☐	☐	☐	☐
We integrate physical activity into other curriculum areas	☐	☐	☐	☐
We use physical activity as a punishment for bad behavior (e.g., withholding recess, administering push-ups or laps)	☐	☐	☐	☐

C6. How do you rate the extent to which teachers at your school act as role models by being physically active?

Very High ☐
High ☐
Moderate ☐
Low ☐
Very Low ☐

C7. What time is recess for the following year levels at your school?

Year 2 _______________ to _____________
Year 4 _______________ to______________
Year 6 _______________ to _____________

C8. What time is lunch for the following year levels at your school?

Year 2 _______________ to _____________
Year 4 _______________ to______________
Year 6 _______________ to _____________

C9. Does your school promote active transportation to and from school in any of the following ways?

	Yes	No
Identify safe routes to use for walking and cycling to and from school (e.g.,with signs, in newsletters, etc.)	☐	☐
Provide crossing guards at intersections to encourage safe walk-to-school routes	☐	☐
Designate a 'car free zone' to provide safe walking areas around the school	☐	☐
Allow students to bring bicycles on school property	☐	☐
Allow students to bring small wheel vehicles (e.g., rollerblades, scooters, skateboards) on school property	☐	☐
Encourage the use of helmets and safety gear for those who use bicycles and small wheel vehicles to get to school	☐	☐
Organize occasional 'walk to school days' , walking clubs, or programs like 'walking school buses'	☐	☐

SECTION D: THE NUTRITION ENVIRONMENT

D1. Rate the level of priority for *nutrition* at your school?

Very Good ☐
Good ☐
Moderate ☐
Poor ☐
Very Poor ☐

D2. Rate the extent to which teachers at your school act as role models by eating healthy foods?

Very Good ☐
Good ☐
Moderate ☐
Poor ☐
Very Poor ☐

D3. Rate the level of support for healthy eating provided by parents at your school?

Very Good ☐
Good ☐

Moderate	☐
Poor	☐
Very Poor	☐

D4. Does your school provide any of the following to promote the sale of healthy food?

	Yes	No
Healthy food choices at a reasonable/subsidized price	☐	☐
Daily healthy eating specials	☐	☐
Healthy eating canteen program (e.g., traffic light labelling system)	☐	☐

D4. During the past 12 months, did your school initiate/continue any of the following activities/programs at your school?

	Yes	No
Offered before school breakfast program	☐	☐
Offered healthy food choices during breakfast program	☐	☐
Offered healthy food choices in the canteen	☐	☐
"Nude Food" program or days	☐	☐
Stopped the sale of junk food	☐	☐
Held junk food free days	☐	☐
Stopped the sale of sugar-sweetened beverages	☐	☐

D5. During the past 12 months, have any of the following items been sold as part of fundraising or events for the school?

	Yes	No
Chocolate or lollies	☐	☐
Other junk food (e.g., chips, popcorn)	☐	☐
Soda pop or fruit drinks that are not 100% juice	☐	☐
Sports drinks	☐	☐
Biscuits, cakes, pastries, or other baked goods that are not low in fat	☐	☐

Fruits or vegetables	☐	☐
100% fruit juice or vegetable juice	☐	☐

D6. Does your school offer any of the following?

	**Yes**	**No**
Cooking classes	☐	☐
Gardening (e.g., growing produce/ school gardens)	☐	☐
Field trips to farms/primary producers	☐	☐
Media literacy on special topics related to healthy eating (e.g., marketing)	☐	☐
Field trips to the local grocery store/farmers' markets	☐	☐

D7. Are students allowed to drink water in the classroom during class time?

Yes ☐
No ☐
Unsure ☐

D8. Are students allowed to eat in the classroom during class time? (brain-food, time set aside before recess and lunch)

Yes ☐
No ☐
Unsure ☐

SECTION E: SCHOOL FACILITIES

E1. Do all students at your school have access to any of the following during school hours?

	Yes	No
Gymnasium	☐	☐
Other large room suitable for physical activity (e.g., multi-purpose room, dance studio)	☐	☐
Fitness room for aerobic and/or strength training	☐	☐
Running track	☐	☐
Outdoor sports field (e.g., football or soccer)	☐	☐
Outdoor paved area (e.g., tennis courts, basketball courts, any paved area that can be used for active games like skipping or hopscotch)	☐	☐
Indoor swimming pool	☐	☐
Change rooms available for use before and after physical activity	☐	☐
Showers available for use after physical activity	☐	☐
Bicycle racks a)	☐	☐
b) that are in a secure area to avoid theft	☐	☐
Grassy playground area	☐	☐
Playground equipment (e.g., climbing structures, swings)	☐	☐

Access to equipment at recess and lunch time (e.g., bats, balls, skipping ropes)	☐	☐

E2. How adequate is the space for indoor play at your school?

Very Good ☐
Good ☐
Moderate ☐
Poor ☐
Very Poor ☐

E3. How adequate is the space for outdoor play at your school?

Very Good ☐
Good ☐
Moderate ☐
Poor ☐
Very Poor ☐

E4. Do all students have access to the following facilities where they can buy foods or drinks?

		Yes	No
Canteen or Cafeteria		☐	☐
Shops/fast food restaurants close to school	≤100 meters	☐	☐
	≤500 meters	☐	☐
	≤1000 meters	☐	☐
Chocolate/lollies and potato chips vending machines		☐	☐
Drinks vending machine (e.g., coke, soft drinks, orange juice)		☐	☐
Milk vending machine (e.g., plain milk, chocolate milk)		☐	☐

E5. Is making a profit an important part of the school canteen?

	Yes	No
a) Profit for the school	☐	☐
b) Profit for the company that runs the canteen	☐	☐

.

E6. Could we have a copy of your school canteen menu?

END OF QUESTIONNAIRE

(Thank you for your time and valuable input)
